# Recent Updates on Local Ablative Therapy Combined with Chemotherapy for Extrahepatic Cholangiocarcinoma: Photodynamic Therapy and Radiofrequency Ablation

**DOI:** 10.3390/curroncol30020166

**Published:** 2023-02-09

**Authors:** Tadahisa Inoue, Masashi Yoneda

**Affiliations:** Department of Gastroenterology, Aichi Medical University, 1-1 Yazakokarimata, Nagakute 480-1195, Japan

**Keywords:** photodynamic therapy, radiofrequency ablation, cholangiocarcinoma, chemotherapy

## Abstract

Although chemotherapy constitutes of the first-line standard therapy for unresectable extrahepatic cholangiocarcinoma, the treatment outcomes are unsatisfactory. In recent years, local ablative therapy, which is delivered to the cholangiocarcinoma lesion via the percutaneous or endoscopic approach, has garnered attention for the treatment of unresectable, extrahepatic cholangiocarcinoma. Local ablative therapy, such as photodynamic therapy and radiofrequency ablation, can achieve local tumor control. A synergistic effect may also be expected when local ablative therapy is combined with chemotherapy. However, it is a long way from being entrenched as an established therapeutic technique, and several unresolved problems persist, including the paucity of evidence comparing photodynamic therapy and radiofrequency ablation. Clinical application of photodynamic therapy and radiofrequency ablation requires sound comprehension and assimilation of the available evidence to truly benefit each individual patient. In this study, we reviewed the current status, issues, and future prospects of photodynamic therapy and radiofrequency ablation for extrahepatic cholangiocarcinoma, with a special focus on their combination with chemotherapy.

## 1. Introduction

The prognosis of extrahepatic cholangiocarcinoma (eCCA), part of a heterogeneous population of biliary tract cancers, remains poor [1,2]. Surgical resection is the only curative treatment method; however, distant metastasis, local invasion, or extensive longitudinal extension may be identified in several cases at the time of diagnosis, which are a contraindication for surgery. Moreover, even if indicated, surgery entails highly invasive procedures, such as hepatectomy and pancreatoduodenectomy, which may not be feasible in older adults and patients with significant underlying diseases. Chemotherapy is the first choice of alternative treatment for unresectable cases. However, the median overall survival (OS) in a phase III trial of gemcitabine plus cisplatin, which is the current standard regimen for biliary tract cancer, was insufficient at 11.7 months [3]. Therefore, breakthroughs are needed for the treatment of eCCA.

In this milieu, endobiliary local ablative therapy performed via the percutaneous or endoscopic approach is a feasible alternative. Because of the direct approachability to the tumor, research on local ablative therapy has progressed, with eCCA as the main target, rather than other biliary tract cancers, such as gallbladder cancer and intrahepatic CCA. Photodynamic therapy (PDT) [4,5] and radiofrequency ablation (RFA) [6,7] are representative local endobiliary treatment modalities. In addition to the local action of ablation, studies have indicated its role in modulating the tumor signaling pathway and immune microenvironment to eliminate the residual tumor and hinder tumor progression [8,9,10,11]. Ablation has also been shown to elicit tumor-specific immune responses by inducing tumor cell death and releasing tumor antigens. These mechanisms of action may act in synergy with systemic chemotherapy. However, although PDT and RFA have yielded promising results, various problems and hurdles persist, preventing their adoption as standard treatments.

In this study, we summarized the evidence on the efficacy of PDT and RFA for treating eCCA, focusing especially on their combination with chemotherapy, and outlined the current status, challenges, and prospects of the two modalities.

## 2. PDT for eCCA

PDT achieves local tumor ablation through the accumulation of a photosensitizer in the region of interest, which is activated by light energy of a specific wavelength and intensity, delivered through an optical fiber that is inserted via an endoscopic or percutaneously to the target area [12]. This generates radical oxygen species capable of inducing localized necrosis of malignant tissues. Ortner et al. [4] conducted a randomized controlled trial to compare the outcomes between 20 patients who underwent biliary stenting with subsequent PDT, and 19 patients who underwent stenting alone. PDT resulted in the prolongation of survival (493 vs. 98 days, *p* < 0.0001) and improved the biliary drainage outcome and quality of life. However, it should be noted that no anti-tumor therapy, including chemotherapy, other than PDT was administered in this study, and only stent placement was performed in the control group. In addition to this study, numerous non-randomized controlled trials have focused on PDT: recent meta-analyses and systematic reviews have shown that using PDT in combination with stent placement significantly improves survival [13,14]. On the other hand, problems such as the complexity of the procedure, high costs, and photosensitivity, which is a characteristic adverse event, have been reported.

## 3. PDT with Chemotherapy for eCCA

Several studies investigated the role of the combination of PDT with chemotherapy for eCCA [15,16,17,18,19] (Table 1). The combination of PDT and chemotherapy yielded a median OS of 17–20 months, which seems longer than what was reported for gemcitabine plus cisplatin alone (OS: 11.2–13.4 months) [3,20,21]. Park et al. [16] conducted a randomized controlled trial to compare PDT plus oral fluoropyrimidine (S-1) and PDT alone, and reported that PDT combined with S-1 significantly prolonged the OS (median 17 vs. 8 months, *p* = 0.005) and progression-free survival (median 10 vs. 2 months, *p* = 0.009). Two retrospective studies compared PDT with chemotherapy and chemotherapy alone. Knüppel et al. [15] reported a higher OS, albeit without a significant difference, in patients who underwent PDT plus chemotherapy with various regimens (median 16.3 vs. 14.5 months, *p* = 0.283), while Gonzalez-Carmona et al. [19] reported a significantly longer OS after the application of PDT in conjunction with various chemotherapeutic regimens (median 20 vs. 10 months, *p* = 0.022). Therefore, we inferred that the additive effect of PDT on chemotherapy-treated patients and that of chemotherapy on PDT-treated patients could be expected. However, as most of the studies were retrospective, patient selection bias in each study was a concern; thus, the results should be interpreted with caution. Furthermore, in these studies, factors that can affect results, such as chemotherapy regimens, number of PDT sessions, and the presence or absence of metastasis, were not standardized. Therefore, it is difficult to draw any conclusions about the combination of PDT and chemotherapy. To obtain sufficient evidence, further well-designed studies, especially derived from randomized controlled trials, are warranted to verify the utility of combination of PDT and chemotherapy.

## 4. Adverse Event of PDT for eCCA

Some adverse events, such as cholangitis, cholecystitis, pancreatitis, liver abscess, peritonitis, sepsis, and phototoxicity, were reported after PDT. The most common adverse event was cholangitis, which is considered less severe and treatable. These adverse events, except phototoxicity, are not intrinsic to PDT, as phototoxicity is a characteristic adverse event associated with PDT. Management of phototoxicity requires special attention, with a recent review reporting an incidence rate of 5.6% [22]. Phototoxicity is one of the major limitations of PDT and may preclude the procedure, considering its indications.

## 5. RFA for eCCA

RFA has been used for the treatment of malignant tumors for a long time. Percutaneous RFA using an electrode needle has been established as a standard treatment modality for some diseases, such as hepatocellular carcinoma [23,24]. The mechanism of action of RFA involves the delivery of thermal energy to the tissue, leading to coagulative necrosis and cell death [25,26,27]. Since the latter half of the 2000s, catheter-type RFA devices consisting of a bipolar catheter with electrodes at the tip were developed and used to treat bile duct lesions [28,29,30]. Two randomized controlled trials were performed to compare RFA plus stent placement versus stent placement alone for eCCA. Yang et al. [31] reported that the mean OS duration was significantly longer in the RFA plus stent group (13.2 vs. 8.3 months, *p* < 0.001), and Gao et al. [32] reported that the median OS was significantly higher in the RFA with stent group (14.3 vs. 9.2 months, *p* < 0.001). However, these results should be interpreted with caution, since the populations of both studies included a mix of locally advanced and distant metastatic diseases, as evidenced by the results of a large-scale retrospective study [33], which showed that the survival extension effect was observed only in eCCA without distant metastasis. Furthermore, Yang et al. excluded patients who received chemotherapy, and Gao et al. included very few patients who received chemotherapy.

## 6. RFA with Chemotherapy for eCCA

Three studies were performed to investigate the combination of chemotherapy and RFA for eCCA [32,33,34] (Table 2). Yang et al. [34] conducted a randomized controlled trial comparing RFA with S-1 and RFA alone for locally advanced eCCA, and reported a significantly higher median OS in the combination group (16.0 vs. 11.0 months, *p* < 0.001). Gonzalez-Carmona et al. [35] conducted a retrospective comparative study for the presence or absence of RFA in patients who underwent gemcitabine-based chemotherapy, and found that the median OS was significantly higher in the RFA–chemotherapy combination group (17.3 vs. 8.6 months, *p* = 0.004). However, a significant difference in the median OS was retained in a subgroup analysis conducted for locally advanced disease (20.9 vs. 12.4 months, *p* = 0.043), while no significant difference was observed for distant metastasis (15.0 vs. 8.6 months, *p* = 0.116). Inoue et al. [36] conducted a retrospective study to examine the effect of RFA in patients treated with the standard regimen of gemcitabine plus cisplatin chemotherapy, and found that the median OS was significantly higher in the RFA–chemotherapy combination group than in the chemotherapy-alone group (17.1 vs. 11.3 months, *p* = 0.017). However, akin to the results of Gonzalez-Carmona et al., a subgroup analysis showed a significant difference in the median OS in patients without distant metastasis (23.1 vs. 16.6 months, *p* = 0.032), whereas no significant difference was observed in patients with distant metastasis (11.4 vs. 8.5 months, *p* = 0.180).

Therefore, we inferred that an additive effect of chemotherapy in patients treated with RFA and the additional effect of RFA in patients treated with chemotherapy could be expected, which appears to be promising, similar to PDT. However, the efficacy of RFA may be limited to locally advanced diseases. In any case, robust evidence is lacking, necessitating detailed investigations into the mechanism of action and accrual of basic research evidence, especially on the systemic effect, including the induction of anti-tumor immunity by endobiliary RFA.

## 7. Adverse Event of RFA for eCCA

Some adverse events, such as cholangitis, cholecystitis, pancreatitis, liver abscess, hemobilia, and sepsis, were reported during and after RFA [6]. RFA is generally regarded as a safe procedure with few serious adverse events, the most common being pancreato-biliary illnesses. In previous comparative studies, no significant difference was observed in adverse event rates between patients treated with or without RFA [6]. However, there have been rare case reports of severe events, including fatal bleeding [37], partial liver infarction [38], biliary tract perforation [39], and hyperkalemia [40]. Additionally, one randomized controlled trial by Gao et al. [32] reported that despite the lack of significant difference in the overall adverse event rate, cholecystitis occurred significantly more frequently in patients treated with RFA than in those not treated with RFA (10.3% vs. 0%, *p* = 0.003). They speculated that ablation near the cystic duct bifurcation could cause edema or damage to the cystic duct opening, leading to cholecystitis.

## 8. Comparison between PDT and RFA for eCCA

Two retrospective studies have compared the efficacy of PDT and RFA for eCCA [35,36] (Table 3). Strand et al. [41] conducted a retrospective study comparing 32 patients who underwent PDT and 16 who underwent RFA, which found no significant difference in the median survival (7.5 vs. 9.6 months, *p* = 0.799). Schmidt et al. [42] conducted a retrospective study that compared 20 patients who underwent PDT and 14 patients who underwent RFA and found that the number of premature stent replacements (<3 months) was significantly higher after the first intervention in the PDT group (65% vs. 29%, *p* < 0.01). Moreover, they stated that post-interventional adverse events tended to occur more frequently in patients with PDT than those with RFA (40% vs. 21%, *p* = 0.277), and phototoxic reactions were observed only in the PDT group.

Currently, it is difficult to judge the superiority or inferiority of RFA and PDT, owing to the scarcity of studies comparing the two modalities and the lack of randomized controlled trials. Additionally, it should be noted that most studies on PDT involved only hilar eCCA. At the same time, RFA tends to include both hilar and distal eCCA; therefore, there is a difference in the tumor populations, which might make interpretation and comparisons difficult. However, the cost difference, premature stent replacement, and presence of phototoxicity would make RFA more favorable than PDT. Additionally, from a technical perspective, RFA is assumed to be simpler than PDT. In any case, well-designed comparative studies, focusing on therapeutic effects, outcomes, adverse events, and costs are needed in the future.

## 9. Future Prospects

Recently, a phase III randomized controlled trial about advanced biliary tract cancer showed that compared with placebo and gemcitabine plus cisplatin, durvalumab combined with gemcitabine plus cisplatin significantly improved OS and showed improvements in prespecified progression-free survival and objective response rate, with safety profiles of the two treatments being similar [43]. Therefore, immunotherapy (combined with chemotherapy) replaces the first-line standard treatment, which is anticipated to become more widespread in clinical practice, even for eCCA [44]. Immunotherapies that trigger the immune system have the potential to enhance the anti-tumor immunity induced by local ablative therapy. Therefore, research facilitating advancement in the application of PDT or RFA in conjunction with immunotherapy is also predicted. Moreover, precision medicine with targeted therapy, such as targeting isocitrate dehydrogenase-1 mutations and the fibroblast growth factor receptor-2 fusions, is now being used for biliary tract cancer [45], although this is mainly applicable to intrahepatic CCA and not as commonly for eCCA. Therefore, research on the combined use of these targeted therapies, and PDT or RFA, is also expected to be conducted in the future.

## 10. Conclusions

This review outlined the current status of PDT and RFA for eCCA, focusing on their combination with chemotherapy. Both methods have the potential to be useful as local therapies, but several aspects remain that require resolution. Moreover, an improvement in devices is essential to enhance the effect of local therapies and make the procedure more straightforward, in order to make the treatments more widespread [46,47]. Further well-designed studies, that include comparisons of the two modalities and accumulate evidence on their outcomes, are warranted.

## Figures and Tables

**Table 1:** Comparative studies regarding photodynamic therapy with chemotherapy for extrahepatic cholangiocarcinoma.

Author	Study Design	Location of Tumor	Stent	Treatment	No. of Patients	Metastasis	No. of PDT Sessions	Median Stent Patency		Median Progression-Free Survival		Median Overall Survival	
Knüppel et al., 2012 [15]	Retrospective	NA	NA	Chemotherapy * + PDT	11	NA	NA	NA	NA	NA	NA	16.3 m	*p* = 0.283
				Chemotherapy *	84	NA	NA	NA		NA		14.5 m	
Park et al., 2014 [16]	Randomized controlled trial	Hilar	PS	S-1 chemotherapy + PDT	21	NA	2.9 (mean)	NA	NA	10 m	*p* = 0.009	17 m	*p* = 0.005
				PDT	22	NA	2.2 (mean)	NA		2 m		8 m	
Hong et al., 2014 [17]	Retrospective	Hilar	PS	Chemotherapy * + PDT	16	31.3%	1 (70.3%) ≥2 (29.7%)	NA	NA	NA	NA	17.9 m	*p* = 0.05
				PDT	58	19.0%		NA		NA		11.1 m	
Wentrup et al., 2016 [18]	Retrospective	Hilar	PS	Chemotherapy * + PDT	33	9.1%	1 (45.5%) 2 (42.4%) 3 (9.1%) 4 (3.0%)	NA	NA	NA	NA	520 d	*p* = 0.021
				PDT	35	2.9%	1 (57.1%) 2 (25.7%) 3 (11.4%) 4 (5.7%)	NA		NA		374 d	
Gonzalez-Carmona et al., 2019 [19]	Retrospective	Hilar, Distal	PS, MS	Chemotherapy * + PDT	36	47.2%	1 (30.6%) ≥2 (69.4%)	NA	NA	NA	NA	20 m	^†^
				PDT	34	14.7%	1 (41.2%) ≥2 (58.8%)	NA		NA		15 m	
				Chemotherapy *	26	69.2%	-	NA		NA		10 m	

PDT, photodynamic therapy; NA, not applicable; m, month; PS, plastic stent; MS, metal stent. * Various regimens were used. ^†^ PDT chemotherapy vs. chemotherapy: *p* = 0.022, PDT chemotherapy vs. PDT: *p* = 0.727, and PDT vs. chemotherapy: *p* = 0.054.

**Table 2:** Comparative studies regarding endobiliary radiofrequency ablation with chemotherapy for extrahepatic cholangiocarcinoma.

Author	Study Design	Location of Tumor	Stent	Treatment	No. of Patients	Metastasis	No. of RFA Sessions	Median Stent Patency		Median Progression-Free Survival		Median Overall Survival	
Yang et al., 2020 [34]	Randomized controlled trial	Distal, hilar	PS	S-1 chemotherapy + RFA	37	0%	3.3 (mean)	6.6 m	*p* = 0.014	12 m	*p* < 0.001	16.0 m	*p* < 0.001
				RFA	38	0%	2.4 (mean)	5.6 m		7 m		11.0 m	
Gonzalez-Carmona et al., 2022 [35]	Retrospective	Distal, hilar	PS	GEM-based chemotherapy + RFA	40	37.5%	1–21	NA	NA	12.9 m	*p* = 0.045	17.3 m	*p* = 0.004
				GEM-based chemotherapy	26	50.0%	-	NA		5.7 m		8.6 m	
Inoue et al., 2022 [36]	Retrospective	Distal, hilar	MS	GEM with cisplatin + RFA	25	48%	1.84 (mean)	10.7 m	*p* = 0.048	8.6 m	*p* = 0.014	17.1 m	*p* = 0.017
				GEM with cisplatin	25	60%	-	5.2 m		5.8 m		11.3 m	

RFA, radiofrequency ablation; PS, plastic stent; m, month; GEM, gemcitabine; NA, not applicable; MS, metal stent.

**Table 3:** Studies comparing photodynamic therapy and radiofrequency ablation for extrahepatic cholangiocarcinoma.

Author	Study Design	Location of Tumor	Stent	Treatment	No. of Patients	Metastasis	No. of PDT or RFA Sessions	Median Stent Patency		Median Progression-Free Survival		Median Overall Survival	
Strand et al., 2014 [41]	Retrospective	Distal, hilar	PS, MS	PDT	32	18.7%	2.09 (mean)	NA	NA	NA	NA	7.5 m	*p* = 0.799
				RFA	16	37.5%	1.19 (mean)	NA		NA		9.6 m	
Schmidt et al., 2016 [42]	Retrospective	Distal, hilar	PS	PDT	20	100%	2 (40%) 3 (25%) 4 (10%) 5 (5%)	NA *	NA *	NA	NA	NA	NA
				RFA	14	93%	2 (50%) 3 (36%) 4 (14%) 5 (7%)	NA *		NA		NA	

PDT, photodynamic therapy; RFA, radiofrequency ablation; PS, plastic stent; MS, metal stent, NA, not applicable; m, months. * The number of premature stent replacements (<3 months) was significantly higher in the PDT group.
